# The Effect of Social Support on Social Media on Asian College Students' Intention to Participate in Physical Activity in the United States

**DOI:** 10.1089/heq.2022.0208

**Published:** 2023-11-20

**Authors:** Ni Zhang, Kele Ding, Chulwoo Park, Jane Vo, Katrina Marie Cantos

**Affiliations:** ^1^ Department of Public Health and Recreation, San Jose State University, San Jose, California, USA.; ^2^ School of Health Sciences, Kent State University, Kent, Ohio, USA.

**Keywords:** Asian, social support, social media, Theory of Planned Behavior

## Abstract

**Background:**

College students who identified themselves as Asians in the United States (i.e., Asian college students) are facing health inequalities and engaging in increasingly low levels of physical activity (PA). Although social support was found to be effective in increasing college students' PA and social media is an important channel for social support for Asian students, few studies have explored how to provide social support through social media interventions to promote Asian students' PA level. Thus, this study aimed to explore the effects of social support on social media on Asian college students' intention to participate in PA based on the theory of planned behavior.

**Methods:**

We conducted an online Qualtrics survey among all undergraduate students at a university on the West Coast of the United States. Among 936 respondents, 337 (36%) were Asian college students. Descriptive analysis, regression models, and mediating effect tests were performed using SPSS 28.

**Results:**

For Asian students, social support on social media has both direct effects and indirect effects through perceived behavioral control (PBC) on their intention to participate in PA.

**Conclusion:**

Future interventions could consider encouraging Asian students to provide support to each other and form support groups using social media to increase their PBC.

## Introduction

Engagement in regular physical activity (PA) is associated with reducing the risk of several chronic conditions, such as cardiovascular disease, diabetes, and obesity.^1^ For college students, it is recommended to engage in a minimum of 150 min of moderate to vigorous PAs every week for substantial benefits.^2^ PA among college students can reduce depression, anxiety, and stress, as well as increase academic performance and cognitive function.^3,4^ PA can also be used to prevent hazardous drinking among college students.^5^ PA patterns during the college years are likely to be sustained into adulthood, which will benefit long-term health.^6^

Given that college students who identified themselves as Asians in the United States (i.e., Asian college students) have one of the highest college attendance rates, changing their PA patterns at college could address the low rates of PA among young Asian adults.^7^ However, Asian college students engage in low levels of PA and nearly half (46.7%) do not engage in moderate to vigorous levels of PA.^8^ Researchers documented that Asian Americans are least likely to meet PA guidelines compared with other racial or ethnic groups.^8^ Other studies have also shown that a large percentage of the Asian student group is not engaging in adequate PA.^9,10^

Asian college students' motivation to participate in PA is influenced by a diverse array of social and cultural factors, including psychological factors (self-efficacy or perceived behavioral control [PBC]), social factors (friends and family), and environmental factors.^11,12^ Colleges are sites for potential intervention as they can allocate resources to promote PA, such as providing information about adopting PA habits, introducing opportunities for PA, and overcoming barriers to PA.^13^ For instance, a campus sports culture with school support for sports teaching, facilities, activities, and competitions is an environmental factor that can help influence PA behaviors.^12^

Social support on social media has been found to be associated with higher intention to participate in PA among college students.^14^ In the general population, social media has proven to be an effective platform to provide social support from family, friends, and colleagues.^15^ For example, a 50-day team-based Facebook application called Active Team, where a group of inactive adult participants were encouraged to take 10,000 steps per day with a pedometer in a team of 3–8 existing Facebook friends who showed social support through peer encouragement, communication of daily tips and advice for increasing PA, and progress monitoring, resulted in high engagement and significantly increased weekly exercise among the participants.^16^

Due to Asian students' potential cultural differences, language barriers, and limited social network, social media is a pivotal communication channel that can provide social support for Asian students seeking to improve PA levels.^17^ Social networking websites and social media applications are key instruments that allow students to share, create, and participate within and outside their social network.

Social media is especially convenient for Asian students, as some students might need it to keep in touch with their acquaintances, friends, or family members from the same ethnic communities who are living far away. With a correlation between lowered PA and increased academic workload, it is imperative to promote solutions on how to use social media as a way to influence and encourage one another to engage in PA and improve overall physical health among Asian college students.^18^

Hence, our study aims to explore the relationship between social support on social media and the intention to participate in PA among Asian college students. According to a study, esteem support from family networks and companionship support from friends directly increase PA behaviors.^19^

In contrast, another study found that social support from family and friends is positively associated with self-efficacy (i.e., PBC), which can then increase PA engagement, showing an indirect relationship between social support and PA engagement.^20^ Few studies have examined how social support on social media is directly or indirectly associated with intention to participate in PA among Asian college students.

Thus, our study fills the gap by examining whether social support on social media is associated with intention to PA directly or indirectly through other constructs in the theory of planned behavior (TPB) among Asian college students. This study has implications for the use of social support on social media as a potential intervention to help increase Asian college students' PA levels.

### Theoretical framework: TPB

The TPB predicts an individual's intention to engage in a specific behavior.^14^ The framework of TPB encompasses four constructs: (1) attitude toward behavior; (2) subjective norms; (3) PBC; and (4) intention.^21^ Similar studies used this theory to predict intent to engage in physical exercise and exercise maintenance among college students, women, elders, and children.^14,15,22^

#### Attitude toward behavior

Attitude toward behavior refers to how an individual approves or disapproves of a particular behavior based on the evaluation of its outcome.^23,24^ There are two types of attitudes: affective attitude and instrumental attitude. According to a report, affective attitude is one's belief about whether the behavior is enjoyable or not, and instrumental attitude is one's belief about whether the behavior is beneficial or harmful to the individual.^23^

Based on TPB, a person's attitude can influence their exercise behaviors. For instance, an individual is more likely to participate in PA if they find exercising to be helpful and enjoyable.^24,25^ Previous studies have found that attitude was one of the strongest predictors of exercise intention.^15,24^

#### Subjective norm

Subjective norm refers to the group attitude and social pressure influencing the individual's desire to perform or not perform a specific behavior.^23^ There are two types of subjective norms^23^: injunctive norms, in which important others around the individual encourage a certain behavior, and descriptive norms, in which the people in the individual's life actively partake in that same behavior.

Although Gomes et al. found that subjective norms affect intention to exercise, many studies have identified that these norms are weak regarding predicting PA behaviors.^26^ For instance, researchers indicated that subjective norms had a lower influence on exercise intentions compared with attitude and PBC.^27,28^

#### Perceived behavioral control

PBC refers to an individual's perception of how easy or difficult it is to perform the behavior.^23^ It assesses how much self-efficacy and manageability a person possesses in dealing with a presented behavior.^24,29^ A cross-sectional study showed that PBC is a significant predictor of PA intentions, where college students' PBC toward exercise accounted for about 36% of the variance.^28^ Many studies have presented similar findings in correlation with PBC.^15,26,28^

### Social support and the TPB

There has been mixed evidence regarding social support's relationship with the constructs of the TPB in predicting intention for PA participation.^24,30^ Studies have suggested that social support has a stronger effect on PA intention than subjective norms do.^31,32^ Conversely, other studies suggest that social support influences intention indirectly through PBC.^15,33–35^

For example, social support for PBC can indirectly affect an individual's perspective about whether they are able to engage in PA behavior and whether it is worth it.^35,36^ Further research is needed to identify the effects of social support on intention to engage in PA using TPB.

**FIG. 1.**
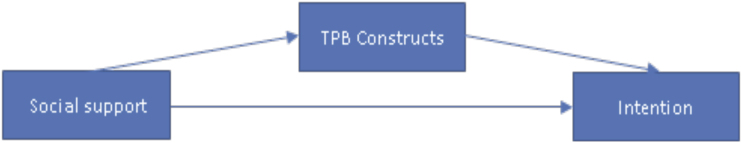
Conceptual model for the association between social media social support and intention to engage in physical activity.

Even though there have been a growing number of studies that examine Asian American college students and their attitude toward participating in PA, there is limited research on the effects of social media support on Asian American college students' intention to engage in PA. The conceptual model of this study is illustrated in Figure 1.

The research question of this study is as follows: How is social support on social media associated with Asian college students' intention to participate in PA directly or indirectly through the TPB constructs (attitude, subjective norm, or PBC)?

## Methods

### Sampling and data collection

The study used a convenience sample. Undergraduate students at a large state university in California were recruited through e-mail from April 2019 to December 2020. E-mail invitations were sent through on-campus student organizations and course instructors.

Participants were asked to fill out an online Qualtrics survey; the survey took 20–30 min to complete. There were 936 respondents in total. One question was asked to all the participants: “What is your race/ethnicity? (Choose all that apply): American Indian or Alaska Native, Asian, Black or African American, Native Hawaiian or other Pacific Islander, or White.” Three hundred thirty-seven students identified themselves as Asian and answered all TPB and social support questions. This percentage of Asian students in the respondents (36%) is consistent with the total percentage of Asian students in the university (31.6%).

The study was approved by the Institutional Review Board of this university.

### Measurement and variables

#### Behavioral intention

Behavioral intention was measured by adapting the scale developed by Courneya et al., with 3 question items on a 7-point ordinal scale.^37^ The scale's Cronbach alpha reportedly ranged from 0.95 to 0.96.^37^

In this study, participants were asked to answer the following questions: (1) “How motivated are you to participate in leisure-time physical activity (LTPA) over the next month?” (1=not at all motivated; 7=extremely motivated); (2) “How strongly do you intend to do everything you can to participate in any LTPA over the next month?” (1=do not intend; 7=strongly intend); and (3) “How committed are you to doing any LTPA over the next month?” (1=not at all committed; 7=completely committed).

Cronbach alpha from our study samples is 0.931. A composite score was calculated by summing the three items. A higher score indicated higher intention to participate in LTPA.

#### Subjective norm

Subjective norms about LTPA were determined from contacts on social networking services (SNSs). Scales developed by Courneya et al. were modified for evaluating subjective norms from the contacts on “this SNS,” referring to the SNS that the participant uses the most.^37^ Since students' social networks enlarge after they join SNSs and befriend people they have never met face to face, the subjective norm was modified to address all the contacts on “this SNS.”

Participants were asked to respond to the statement, “I think that if I were to participate in leisure-time physical activity over the next month, my contacts on this social network site would be…,” on three semantic differential items: disapproving–approving, unsupportive–supportive, and discouraging–encouraging. Each item was rated on a 5-point scale ranging from 1 to 5.

Cronbach alpha from our study samples is 0.936. The composite score was calculated by summing the three items. Higher scores indicate students' perceptions that their contacts on SNSs would be more approving of their participation in LTPA.

#### Attitude

Attitude was measured by two subscales: the affective attitude scale and the instrumental attitude scale with a 5-point semantic differential scale.^34,38^ The affective attitude scale included questions about their emotional ratings of LTPA.

Respondents were asked to rate “To me, participating in leisure-time physical activities is” boring (1) to interesting (5); unenjoyable (1) to enjoyable (5); unpleasant (1) to pleasant (5); bad (1) to good (5); and undesirable (1) to desirable (5). For the instrumental attitude scale, respondents were asked to rate whether they believed participating in LTPA was harmful (1) to beneficial (5); useless (1) to useful (5); weak (1) to strong (5); passive (1) to active (5); and foolish (1) to wise (5).

Cronbach alpha from our study samples on this section is 0.936. The analysis first calculated a sum score for each of the subscales and then took the mean of the two sums as the attitude score for this study.

#### Perceived behavioral control

PBC among undergraduate college students was measured by using an existing instrument used by Okun et al.^34,38^ Respondents were asked to indicate the extent to which they agreed with the following two items for LTPA: “I have the resources required to engage in leisure-time physical activity” and “It is easy for me to engage in leisure-time physical activity,” on a 5-point Likert scale that ranged from 1 (strongly disagree) to 5 (strongly agree).

Cronbach alpha from our study samples is 0.734. The sum score of these two items was used in this analysis. A higher score indicated higher PBC.

#### Social support for LTPA on social media

Social support for LTPA on social media was measured by using a modified version of the Social Influence on Physical Activity Questionnaire developed by Chogahara.^39^ Social support in this 5-point Likert scale, ranging from 1 (never) to 5 (very often), was measured in 3 subscales with 5 question items: (1) companionship support, which refers to participating in an LTPA with another person; (2) informational support, which provides positive information regarding LTPA; and (3) esteem support, which refers to encouragement.

Participants were asked to indicate how often their contacts have said or done (using social media that they use most often) what is described in the following items in the last 12 months. An example item for the informational support subscale is “informed you about the expected positive effects of a leisure-time physical activity on your health”; and an example item for esteem support is “complimented your good skill in a leisure-time physical activity.” All question items were worded in the same direction.

Cronbach alpha from our study samples is 0.965. Sum scores of each subscale were first obtained and then a mean was taken to represent the social support score. Higher scores indicated more social support for LTPA.

#### Demographic variables

Demographic variables, including age, gender, race, first generation of college degree, and school year, were used in this study.

### Data analyses

Information about race and ethnicity was collected through a set of 15 check-all-that-apply questions asking whether they are Hispanic, White, Black, American Indian or Alaska Native, Asian Indian, Chinese, Filipino, Japanese, Korean, Vietnamese, Hawaiian, Guamanian, Samoan, or other Asians. Those who checked Asian Indian, Chinese, Filipino, Japanese, Korean, Vietnamese, and/or other Asians were chosen as study subjects.

Descriptive analyses were used to inspect the distribution of included demographic variables, TPB variables, and social support composite variables. Mean scores of the TPB and social support composite variables were compared between demographic categories and across country origins using a *t*-test and analysis of variance test. To examine the relationship between social support and LTPA behavioral intention, the mediating effects of normative beliefs, behavior control, and attitude in the relationship were used. In addition, a Matrix macro program was employed with a bootstrap test option.

In the mediation model tests, the TPB variables, attitude, normative belief, and behavior control, were used as mediators; social support was used as the independent variable; and behavioral intention was used as the dependent variable. Gender, age, first generation, and years in school were variables of control. We first tested the initial model without the three mediators in the model and then we tested the full model by including the three mediator variables.

All statistical analyses were conducted using SPSS, version 28. The significance level threshold was set as equal to or less than 0.05.

## Results

Of the 337 Asian respondents, 80 (23.7%) identified themselves as Filipino, 79 (23.4%) as Vietnamese, and 69 (20.5%) as Chinese. A majority of respondents are females (64.4%), not the first generation of college students (57%), and in their third or fourth year in undergraduate programs (65.6%). Mean age is 23.1 (ranging from 18 to 45) years with a standard deviation of 4.28 years.

The bivariate analysis (Table 1) revealed that female students had a higher score in attitude and lower score in PBC than male students (22.3 vs. 21.3 and 7.3 vs. 7.8, respectively). For the year in school, a significant difference was found for the social support variable, where first- or second-year students had a significantly higher score than the fifth year or more category (14.6 vs. 11.0).

**Table 1.** The levels of behavioral intention, subjective norms, attitudes, perceived behavioral control, and social support among Asian college students surveyed at a large state university in California between April 2019 and December 2020, by demographic characteristics

Variable	Category (***n***)	Intention, mean (SD)	Subjective norm, mean (SD)	Attitudes, mean (SD)	PBC, mean (SD)	SS, mean (SD)
Gender	Male (117)	15.1 (4.77)	13.0 (2.28)	21.3 (3.74)^*^	7.8 (1.81)^*^	13.1 (5.48)
	Female (217)	15.2 (3.85)	13.4 (2.07)	22.2 (3.18)	7.3 (1.92)	12.7 (4.61)
First generation	Yes (133)	14.6 (4.70)	13.2 (2.30)	21.9 (3.52)	7.3 (1.89)	12.5 (4.77)
	No (192)	15.5 (3.72)	13.3 (2.06)	21.9 (3.29)	7.6 (1.88)	13.2 (5.09)
School year	First or second year (22)	15.3 (4.38)	13.1 (2.21)	22.4 (2.56)	7.9 (2.01)	14.6 (5.47)^**^
	Third year (101)	15.1 (3.98)	13.1 (2.25)	21.3 (3.76)	7.4 (1.85)	13.3 (4.34)
	Fourth year (120)	14.8 (4.27)	13.5 (2.00)	22.6 (3.20)	7.5 (1.96)	12.6 (4.90)
	Fifth year or more (46)	15.9 (4.27)	13.1 (2.27)	21.4 (3.75)	7.5 (1.97)	11.0 (5.14)^**^
	Transferred (48)	15.2 (4.33)	13.2 (2.15)	21.8 (3.11)	7.4 (1.74)	13.6 (5.20)
Country of origin	Indian (27)	15.8 (3.20)^**^	12.4 (2.61)	20.8 (3.83)	7.9 (1.80)	15.3 (4.82)
	Chinese (69)	13.8 (4.01)	13.1 (2.15)	21.8 (3.10)	7.1 (1.85)	11.8 (4.59)
	Filipino (80)	15.9 (3.79)	13.7 (1.87)	22.5 (3.36)	7.5 (2.10)	13.2 (4.68)
	Japanese (8)	12.4 (4.34)	12.9 (2.31)	20.1 (4.29)	7.4 (1.69)	12.9 (3.77)
	Korean (4)	16.3 (2.50)	12.6 (1.60)	22.3 (3.28)	7.5 (1.91)	15.3 (7.00)
	Vietnamese (79)	14.6 (4.53)	13.2 (2.14)	22.1 (3.52)	7.4 (1.82)	12.6 (5.24)
	Other Asians (39)	16.4 (4.42)	13.5 (2.31)	21.5 (3.65)	7.8 (1.87)	12.7 (5.40)
	Multiethnicities (31)	15.8 (4.34)	13.1 (2.05)	22.1 (3.21)	7.6 (1.82)	12.8 (4.39)
Ethnicity	Asian (426)	15.2 (4.21)	13.5 (2.23)	43.7 (6.94)	7.5 (1.89)	38.3 (15.02)
	Hispanic/Latino (96)	15.7(3.65)	13.5 (2.33)	45.2 (5.87)	7.3 (2.24)	36.3 (14.97)
	White (193)	15.9 (4.01)	13.5 (2.28)	45.0 (5.97)	8.0 (2.01)	36.5 (14.84)
	African American (36)	15.4(4.40)	13.0 (3.16)	43.2 (8.49)	7.5 (2.10)	42.3 (14.90)
	Others (65)	16.7 (4.49)	13.8 (2.47)	45.8 (6.59)	7.8 (2.13)	38.1 (17.28)

^*^ *p* value is 0.05 or less; ^**^p value is 0.01 or less.

PBC, perceived behavioral control; SD, standard deviation; SS, social support.

Table 2 shows the direct effects of all independent variables on intention. Social support was a significant predictor of behavioral intention (coefficient β=0.18, *p*=0.001). Both attitude (β=0.2989, *p*=0.001) and PBC (β=0.878, *p*=0.001) made a significant contribution to the behavioral intention.

**Table 2.** Coefficients of direct effects of social support, subjective norm, attitude, and perceived behavioral control on behavioral intention of engaging in physical activities among Asian college students surveyed at a large state university in California between April 2019 and December 2020 (*n*=323)

	β	Intention		
		SE	***t***	***p***
SS	0.1803	0.038	4.7404	0.001
Subjective norm	−0.0025	0.1007	−0.0247	0.9803
Attitude	0.2989	0.0648	4.6103	0.001
PBC	0.878	0.1046	8.3971	0.001
Gender	0.3442	0.3942	0.8733	0.3831
Age	−0.0479	0.0426	−1.1234	0.2621
First-generation college student	0.6539	0.3449	1.8956	0.0589
Years in school	0.2025	0.1604	1.263	0.2075

β=coefficient. Covariates controlled in the model include age, gender, first-generation college student, and years in school.

SE, standard error.

Table 3 shows the indirect effect of social support on the behavioral intention of engaging in PA.

**Table 3.** Coefficients of indirect effects of social support on behavioral intention of engaging in physical activities among Asian college students surveyed in a large state university in California between April 2019 and December 2020 (*n*=323)

TPB constructs	Intention			
	β	SE (boot)	Confidence interval	
Subjective norm	0	0.0031	−0.0069	0.0064
Attitude	0.0158	0.0121	−0.0061	0.0422
PBC	0.0502	0.0199	0.014	0.0925

β=coefficient.

TPB, theory of planned behavior.

## Discussion

Consistent with previous research, our study found that Asian college students had the lowest intention to participate in PA among all ethnic groups in the United States. In addition, we found that Asian students had the second lowest attitude toward PA among all ethnic groups, followed by African American students (Table 1).

Given that Asian students have one of the highest college attendance rates, colleges and universities are ideally situated to address the low rates of PA among young Asian adults in the United States. Our study shows that colleges and universities could potentially use social support through social media to promote the PA levels among Asian college students.

We found social support on social media to have both direct and indirect effects on Asian college students' intention to participate in PAs. First, we found that social support as well as attitude and PBC had direct effects on Asian college students' intention to participate in PAs. Our results showed that social support acted as a strong predictor for behavioral intention. This was consistent with Van Luchene and Delens's study, where they discovered that social support specific to PA was related to the practice of PA among college and university students.^20^

Second, we found that social support had an indirect effect on Asian students' intention to participate in PA through PBC, which was consistent with other studies.^15,33,35^ This means that more social support is associated with increased PBC, which can lead to higher intention to participate in PA among Asian college students.

Third, we found that social support did not have an indirect effect on Asian students' intention to participate in PA through attitude, which was inconsistent with the findings in another study, where the majority of the participants were White.^15^ This means that for Asian college students, social support might not be able to influence their attitudes toward PA, which can impact their intention to participate in PA.

The study results shed light on future public health intervention and practice design tailored toward Asian college students to focus on social support's role in increasing their PBC instead of changing their attitude toward PA in the United States.

Due to potential cultural differences, language barriers, limited social networks, and acculturation, social support plays an important role in increasing Asian college students' PBC or confidence about performing the behavior, which in turn increases the intention to participate in PAs.

For example, they might need to find people from the same community, speaking the same language, to perform the PA together. We could provide companionship support to organize sports activities on social media. At the same time, we could also form social support groups for Asian college students on social media to share information about how to perform or improve certain PAs to increase their PBC.

Social media can be used as leverage to encourage peers to engage in physical exercise, such as creating specialized online support groups and discussion boards where individuals can give each other feedback and advice, recommending fitness apps for PA tracking and guidance, providing accessible PA locations, and having a buddy pass system where participants can bring a partner with them each time they exercise.

This study has several limitations. First, the main limitation is regarding the sample. We collected the surveys using convenience sampling. Mass e-mail to all the students at the university was not allowed, so e-mails were sent out to students by individual instructors and student organizations. The Asian student sample was selected from all the students' responses in a university in the United States. One needs to be cautious in generalizing the results to the larger Asian population in the United States. Future study can recruit Asian students or the Asian population in the United States.

Second, the survey was sent out in English to students of all ethnicities. Future research can translate the survey into different Asian languages to tailor it to Asian students in the United States who prefer to answer the survey in their own ethnic language. Third, self-report questionnaires might cause recall bias.

Fourth, all the participants who identified themselves as Asian were included in the study. No information about their immigration status was collected. The sociocultural attitudes and beliefs are likely to differ between Asian students who immigrated to the United States by themselves (first-generation immigrants) and Asian students who are second- or third-generation immigrants. Future study can collect the immigration status of Asian students and include it as a covariate in the analysis.

This study confirmed the need to tailor interventions on social media to promote health equity regarding PA among Asian college students. We found that Asian college students need direct social support on social media as well as indirect social support through a group on social media that can improve their PBC and make them feel confident to participate in PA.

## Abbreviations Used

LTPA: leisure-time physical activity
PA: physical activity
PBC: perceived behavioral control
SD: standard deviation
SE: standard error
SNS: social networking service
SS: social support
TPB: theory of planned behavior
